# PANCREATIC NEUROENDOCRINE TUMORS: CLINICOPATHOLOGICAL ANALYSIS AND POSTOPERATIVE SURVIVAL FOLLOWING SURGICAL RESECTION-A DECADE OF EXPERIENCE AT A TERTIARY UNIVERSITY HOSPITAL

**DOI:** 10.1590/S0004-2803.24612025-089

**Published:** 2026-10-26

**Authors:** Mariana Martins Sanches FERREIRA, Philipe Franco do Amaral TAFNER, Martinho Antonio GESTIC, Murillo Pimentel UTRINI, Francisco CALLEJAS-NETO, Everton CAZZO

**Affiliations:** * Universidade Estadual de Campinas (UNICAMP), Faculdade de Ciências Médicas, Campinas, SP, Brasil.

**Keywords:** Neuroendocrine tumors, pancreas, pancreatectomy, surgery, prognosis, Tumores neuroendócrinos, pâncreas, pancreatectomia, procedimentos cirúrgicos, prognóstico

## Abstract

**Background:**

Pancreatic neuroendocrine tumors (PNETs) are rare neoplasms of the digestive system, with variable prognoses and biological behaviors. Surgical resection remains the mainstay of treatment. Objective: To evaluate the clinicopathological characteristics and surgical outcomes of patients with PNETs who underwent resection at the Hospital de Clínicas, UNICAMP.

**Methods:**

This is a retrospective cohort study including 24 patients who underwent surgical resection between 2013 and 2023. Clinical, demographic, histopathological, and survival data were analyzed. The primary outcome was overall survival.

**Results:**

The mean age was 53.9 ± 14.4 years, with a predominance of female patients (66.7%). Most tumors were nonfunctioning (79.1%), with abdominal pain (79.2%) and weight loss (50%) being the most common symptoms. The surgical procedures performed were distal pancreatectomy (54.2%), pancreaticoduodenectomy (41.6%), and enucleation (4.2%). Histological classification revealed 62.5% grade 2 tumors, 33.3% grade 1, and 4.2% MiNEN. Mean overall survival was 70.4 ± 58 months. There was a trend toward longer survival in grade 1 tumors (98 ± 68.1 months) compared to grade 2 (59.1 ± 49.5 months; *P*=0.06). Postoperative mortality was 8.3%, associated with pancreaticoduodenectomy.

**Conclusion:**

Histological grading remains a key prognostic factor in PNETs. Surgical resection remains the cornerstone of treatment. Multicenter studies are necessary to validate these findings on a larger scale.

## HIGHLIGHTS

• Most resected pancreatic neuroendocrine tumors (PNETs) were classified as grade 2 (62.5%), with a significant proportion of cases demonstrating intermediate proliferative activity (Ki-67 index 1-10%). This reflects a surgical cohort selected for operability and emphasizes the need for tailored management strategies in this tumor grade range.

• Most tumors were nonfunctioning (79.1%) and presented with nonspecific symptoms such as abdominal pain (79.2%) and weight loss (50%). These clinical features contributed to diagnostic delays and highlight the insidious nature of PNETs compared to more symptomatic functioning variants.

• Surgical resection remained the primary therapeutic approach, with a mean overall survival of 70.4 months. There was a trend toward longer survival in patients with grade 1 tumors, underscoring the prognostic significance of tumor grade.

## RESUMO

**Contexto:**

Os tumores neuroendócrinos pancreáticos (TNEPs) são neoplasias raras do sistema digestivo, apresentando prognóstico e comportamento biológico variáveis. A ressecção cirúrgica permanece como o principal tratamento.

**Objetivo:**

Avaliar as características clinicopatológicas e os desfechos cirúrgicos de pacientes com TNEPs submetidos à ressecção no Hospital de Clínicas da UNICAMP.

**Métodos:**

Estudo de coorte retrospectivo que incluiu 24 pacientes submetidos à ressecção cirúrgica entre 2013 e 2023. Foram analisados dados clínicos, demográficos, histopatológicos e de sobrevida. O desfecho primário foi a sobrevida global.

**Resultados:**

A média de idade foi de 53,9 ± 14,4 anos, com predomínio do sexo feminino (66,7%). A maioria dos tumores era não funcionante (79,1%), sendo dor abdominal (79,2%) e perda ponderal (50%) os sintomas mais frequentes. Os procedimentos cirúrgicos realizados foram pancreatectomia distal (54,2%), duodenopancreatectomia (41,6%) e enucleação (4,2%). A classificação histológica revelou 62,5% de tumores grau 2, 33,3% grau 1 e 4,2% MiNEN. A sobrevida global média foi de 70,4 ± 58 meses. Observou-se tendência a maior sobrevida nos tumores grau 1 (98 ± 68,1 meses) em comparação aos tumores grau 2 (59,1 ± 49,5 meses; *P*=0,06). A mortalidade pós-operatória foi de 8,3%, associada à duodenopancreatectomia.

**Conclusão:**

O grau histológico permanece como importante fator prognóstico nos TNEPs. A ressecção cirúrgica continua sendo a base do tratamento. Estudos multicêntricos são necessários para validar esses achados em populações maiores.

## INTRODUCTION

Pancreatic neuroendocrine tumors (PNETs) are rare neoplasms arising from the islet cells of Langerhans in the pancreas. They account for approximately 1-2% of all pancreatic tumors and exhibit a wide spectrum of biological behaviors and clinical presentations^1^. PNETs are classified as either functioning, when they secrete biologically active hormones, or nonfunctioning, when there is no significant hormonal secretion. Functioning PNETs include insulinomas, gastrinomas, glucagonomas, and VIPomas, with insulinomas being the most prevalent subtype^2^.

Most PNETs are diagnosed incidentally due to the increasing use of cross-sectional imaging modalities such as computed tomography (CT), magnetic resonance imaging (MRI), and endoscopic ultrasonography with fine needle aspiration (EUS-FNA). These advancements have contributed to the earlier detection of asymptomatic or minimally symptomatic tumors^3^.

Historically referred to as carcinoid tumors, PNETs are now more precisely classified due to advances in histopathology and immunohistochemistry. The World Health Organization (WHO) currently categorizes these tumors based on proliferative activity, using the Ki-67 labeling index and mitotic count. This classification stratifies tumors into grade 1 (G1), grade 2 (G2), grade 3 (G3), and poorly differentiated high-grade neuroendocrine carcinomas^4^. Differentiating between well-differentiated and poorly differentiated neoplasms is essential, as it has significant implications for both prognosis and therapeutic management.

Surgical resection remains the cornerstone of treatment, particularly for functioning tumors or lesions with malignant potential. Nonetheless, conservative approaches such as active surveillance and enucleation may be appropriate for small, nonfunctioning, well-differentiated tumors in selected patients^5^. The clinical significance of PNETs, coupled with the limited data available from Brazilian institutions, prompted this retrospective study aimed at evaluating the clinicopathological features and surgical outcomes of patients with PNETs treated at a university-based referral center.

## METHODS

### Study design

This is an observational, retrospective, single-center study with a historical cohort design, conducted at the Hospital de Clínicas da UNICAMP, a tertiary referral center. Patients with a confirmed histopathological diagnosis of pancreatic neuroendocrine tumors (PNETs) who underwent surgical intervention between January 2013 and December 2023 were included. The project was approved by the institution’s Research Ethics Committee.

### Study population

The inclusion criteria were age 18 years or older, presence of pancreatic neuroendocrine neoplasia confirmed by histopathological examination, and surgical resection. Patients with metastatic neuroendocrine tumors not primarily resected or with incomplete data in the medical records were excluded. Patients were identified through the electronic surgical records system, which was used to retrieve all pancreatic resections performed between 2013 and 2023, including pancreaticoduodenectomy, gastropancreaticoduodenectomy, distal pancreatectomy, body-tail pancreatectomy, total pancreatectomy, and pancreatic enucleation. From this cohort, patients with pancreatic neuroendocrine tumors were identified based on the histopathological analysis of the resected surgical specimens.

### Study variables

The variables analyzed included demographic (age, sex, BMI), clinical (symptoms, presence of hormonal syndrome), surgical (type of procedure, surgical time, complications) and histopathological (Ki-67 index, mitotic index, WHO differentiation grade) data. The primary outcome was overall survival, defined as the time between the date of diagnosis and death or last follow-up. Surgical mortality (death within 30 days after surgery or during hospitalization) was considered a secondary outcome.

### Statistical analysis

Statistical analysis was performed using R software version 4.2.0. Survival was estimated by the Kaplan-Meier method, and differences between groups were assessed by the log-rank test. Differences between categorical variables were tested with Fisher’s exact test or chi-square test, and continuous variables by Student’s t-test or Mann-Whitney test. Values of *P*<0.05 were considered statistically significant.

## RESULTS

A total of 24 patients with pancreatic neuroendocrine tumors (PNETs) who underwent surgical resection were included in the analysis. The mean age was 53.9 ± 14.4 years, with a predominance of female patients (66.7%). The mean body mass index (BMI) was 27.3 ± 5.3 kg/m². The most frequently reported symptoms were abdominal pain (79.2%), weight loss (50%), jaundice (20.8%), and nausea (12.5%). Functioning syndromes were identified in five patients (20.8%), four of whom had insulinoma and one had a VIPoma.

The surgical procedures performed included distal pancreatectomy (54.2%), pancreaticoduodenectomy (41.6%), and pancreatic enucleation (4.2%). According to the World Health Organization (WHO) classification, tumors were classified as grade 1 in 33.3% of cases, grade 2 in 62.5%, and MiNEN (mixed neuroendocrine-non-neuroendocrine neoplasm) in 4.2%. No cases were classified as grade 3. The Ki-67 proliferation index ranged from 1-10% in 83.4% of patients, while the mitotic index was less than 5 mitoses per 10 high-power fields in 50% of cases. The mitotic index was not recorded in six patients (25%).

The overall surgical mortality rate was 8.3%, with two postoperative deaths following pancreaticoduodenectomy. The mean overall survival from diagnosis was 70.4 ± 58 months. Patients with grade 1 tumors demonstrated a mean survival of 98 ± 68.1 months, compared to 59.1 ± 49.5 months for those with grade 2 tumors, showing a trend toward statistical significance (*P*=0.06).

Table 1 summarizes the main clinical and surgical characteristics of the cohort, including demographic distribution, presenting symptoms, surgical approach, and histopathological findings. Figure 1 presents the Kaplan-Meier survival curve comparing overall survival between grade 1 and grade 2 tumors.

**TABLE 1:** Main characteristics of the study population.

N	24
Age (years)	53.9±14.4
Gender	Female: 16 (66.7%)
	Male: 8 (33.3%)
BMI (kg/m2)	27.3±5.3
Symptoms	Weight Loss: 12 (50%)
	Abdominal Pain: 19 (79.2%)
	Jaundice: 5 (20.8%)
Hormone hypersecretion syndrome (N=5)	Insulin hypersecretion: 4 (16.7%)
	VIP hypersecretion: 1 (4.2%)
Operation - N (%)	Body-tail or Distal Pancreatectomy: 13 (54.2%)
	Enucleation: 1 (4.2%)
	Pancreatoduodenectomy: 10 (41.6%)
Histological Grade - N (%)	G1: 8 (33.3%)
	G2: 15 (62.5%)
	G3: 0
	MiNEN: 1 (4.2%)
Ki-67 (%)	<1%: 3 (12.5%)
	1-5%: 10 (41.7%)
	5-10%: 10 (41.7%)
	>10%: 1 (4.1%)
Mitotic index (mitoses per 10 high-Power field)	<5: 12 (50%)
	5-10: 3 (12.5%)
	>10: 3 (12.5%)
	Not evaluated: 6 (25%)
Operative mortality - N (%)	2 (8.3%)
Postoperative Survival (months)	70.4 ± 58

N: number of individuals. MiNeN: mixed neuroendocrine-non-neuroendocrine. Neoplasm. VIP: vasoactive intestinal polypeptide. BMI: body mass index.

**FIGURE 1:**
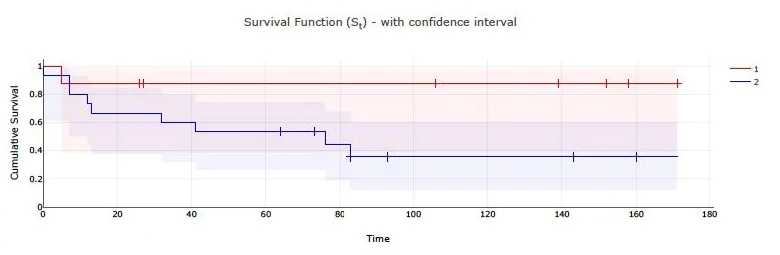
Comparison of long-term survival of patients undergoing resection of pancreatic neuroendocrine tumors according to histological grade (G1 vs G2).

## DISCUSSION

The findings of this study underscore the clinical and biological heterogeneity of pancreatic neuroendocrine tumors (PNETs), highlighting their low incidence, insidious clinical presentation, and histopathological diversity. This heterogeneity poses significant challenges in both diagnosis and treatment, as these neoplasms range from slow-growing, indolent tumors to highly aggressive malignancies.

The mean patient age in this cohort (53.9 years), with a female predominance (66.7%), aligns with national series such as those by Figueira et al.^6^ and Gavrilescu et al.^7^, which reported comparable age distributions. While some studies describe a slight male predominance, the overall literature remains inconsistent with respect to sex, possibly reflecting regional differences and referral patterns.

The frequency of functioning tumors (20.8%) is consistent with previously published Brazilian data. As reported by Belotto et al.^8^, insulinomas are the most common functional subtype, a finding mirrored in the present study. These tumors tend to be diagnosed earlier due to clinical manifestations of hormone hypersecretion. In contrast, nonfunctioning PNETs (NF-PNETs)-which represented the majority of cases in this cohort-are typically asymptomatic and detected only at more advanced stages. This distribution reflects international trends and underscores the importance of clinical vigilance, especially in individuals with hereditary tumor syndromes^1^ ^-^ ^3^.

Most patients presented with nonspecific symptoms, the most common being abdominal pain (79.2%). Although frequent, this symptom lacks specificity and may contribute to diagnostic delays, given its overlap with various benign gastrointestinal disorders. In PNETs, pain can be attributed to tumor growth and local compression of structures such as the pancreatic duct, vascular elements, and bowel loops. Jaundice was reported in only 21% of cases-an important distinction from pancreatic ductal adenocarcinoma, where obstructive jaundice is often an early sign. This contrast likely stems from the expansive, non-infiltrative nature of PNETs, in contrast to the infiltrative behavior of adenocarcinomas, reinforcing the need for a high index of suspicion in clinical practice^4^.

Histopathological evaluation confirmed the predominance of grade 2 tumors, followed by grade 1 (33.3%) and one case of MiNEN (4.2%), a rare neoplasm containing both neuroendocrine and non-neuroendocrine components. These findings are consistent with other surgical series and emphasize the intermediate biological aggressiveness typical of resected cases. The predominance of grade 2 tumors (62.5%) may reflect the patient selection typical of tertiary care centers, where individuals with intermediate-risk disease are more likely to be offered surgical intervention. This distribution may also explain the moderate overall survival observed (70.4 ± 58 months). The absence of grade 3 tumors likely indicates selection bias, as these more aggressive neoplasms are commonly managed with systemic, non-surgical therapies.

Regarding proliferative indices, most tumors fell within an intermediate Ki-67 range: 41.7% of cases had values between 1-5%, and another 41.7% between 5-10%. Only three patients (12.5%) had a Ki-67 below 1%, indicating a more indolent course, while one case (4.1%) exceeded 10%, approaching the grade 3 threshold. These patterns reinforce the predominance of tumors with intermediate proliferation, which are often associated with unpredictable biological behavior. Regarding the mitotic index, half of the patients (50%) had fewer than 5 mitoses per 10 high-power fields (HPF), consistent with grade 1 tumors. An additional 12.5% had between 5-10 mitoses, and another 12.5% exceeded 10 mitoses per 10 HPF, which could theoretically indicate more aggressive behavior. However, in 25% of the specimens, the mitotic index was not reported, representing a methodological limitation and emphasizing the need for standardization in histopathological reporting to ensure accurate prognostic stratification.

Taken together, the histopathological findings highlight the importance of an integrated assessment of tumor grade, Ki-67 index, and mitotic index. These parameters are not only essential for tumor classification and prognostication but also for guiding therapeutic decisions, particularly as conservative management approaches gain traction for selected patients. Current guidelines recommend non-operative surveillance for nonfunctioning PNETs smaller than 2 cm with presumed low-grade features. This strategy, aimed at minimizing overtreatment in patients with indolent disease, likely influenced the findings of this study. Many of these tumors may not have been resected, which could explain the underrepresentation of grade 1 tumors and the absence of grade 3 tumors in the surgical sample. Similarly, the relatively low number of functioning tumors may reflect early management via enucleation or, in more advanced cases, contraindications to surgery due to metastasis or comorbidities. Therefore, the cohort likely represents a selected group of surgically eligible patients, a factor that must be considered when interpreting the histopathological and survival data^9^.

The observed survival difference between histologic grades-98 months for grade 1 versus 59.1 months for grade 2 (*P*=0.06) -although not statistically significant, reinforces the prognostic relevance of tumor differentiation as defined by the World Health Organization (WHO) classification. This trend toward longer survival in grade 1 tumors aligns with multiple international studies, and the absence of statistical significance may simply reflect the study’s limited sample size, underscoring the need for larger, multicenter investigations.

The postoperative mortality rate of 8.3%, which occurred exclusively in patients undergoing pancreaticoduodenectomy, reflects the technical complexity and elevated morbidity associated with this procedure. This finding is consistent with the literature, including the study by Souche et al.^10^, which identified pancreaticoduodenectomy as the highest-risk pancreatic resection, particularly in patients with comorbidities or locally advanced tumors.

In recent years, there has been growing interest in the role of adjuvant and neoadjuvant therapies, particularly in patients with borderline resectable or metastatic disease. Peptide Receptor Radionuclide Therapy (PRRT) has emerged as a promising option for well-differentiated (G1/G2) tumors expressing somatostatin receptors^11^. The most compelling evidence for PRRT comes from the NETTER-1 trial, which demonstrated a significant improvement in progression-free survival with ^177^Lu-DOTATATE compared to high-dose octreotide therapy^12^. In addition to PRRT, somatostatin analogs such as octreotide and lanreotide remain first-line therapies for symptom control and tumor stabilization in patients with advanced disease. Targeted therapies-including everolimus and sunitinib-have also shown efficacy in progressive cases, as supported by randomized clinical trials^11^. Accordingly, the institutional experience reported here, though limited in scale, emphasizes the need for individualized treatment strategies based on tumor biology, disease extent, and overall patient status. The integration of surgical resection with modern systemic therapies broadens the therapeutic landscape and may significantly improve outcomes in this complex patient population^13^ ^,^ ^14^.

This study has several limitations that should be acknowledged. As a retrospective, single-center analysis with a relatively small sample size, the findings are subject to both statistical limitations and restricted generalizability. Missing data-such as the mitotic index in 25% of cases-may have led to inaccuracies in tumor grading. Additionally, selection bias likely influenced the cohort composition, as small, low-risk tumors managed with active surveillance were excluded. Variability in surgical technique and follow-up protocols may also have affected outcomes. Nevertheless, this study has notable strengths: it represents one of the few institutional Brazilian series focusing exclusively on resected PNETs, employing updated histopathological classifications and collecting comprehensive clinical data. Survival analysis stratified by tumor grade contributes valuable insight into the real-world behavior of these tumors and enriches the national understanding of a rare and underexplored neoplasm in surgical oncology.

## CONCLUSION

This study demonstrated that pancreatic neuroendocrine tumors (PNETs) surgically treated at the Hospital de Clínicas da UNICAMP exhibit clinical and pathological characteristics consistent with those reported by major reference centers. The predominance of grade 2 tumors, the proportion of functioning syndromes, and the anatomical tumor distribution align with findings in the broader literature, supporting the external validity of the results. Although limited by a small sample size, the study identified a trend toward improved survival in patients with grade 1 tumors, reinforcing the prognostic value of tumor differentiation. Surgical resection remains the cornerstone of treatment, offering acceptable survival outcomes. However, to optimize therapeutic strategies-particularly in borderline cases and tumors with intermediate biological behavior-multicenter studies involving larger cohorts are essential for the development of more standardized management protocols.

## Data Availability

data are available from the corresponding author upon reasonable request.

## References

[1] Strosberg JR (2024). Classification, epidemiology, clinical presentation, localization, and staging of pancreatic neuroendocrine neoplasms. UpToDate.

[2] Ma ZY, Gong YF, Zhuang HK (2020). Pancreatic neuroendocrine tumors: a review of serum biomarkers, staging, and management. World J Gastroenterol.

[3] Souche R, Coignac A, Dupuy M (2020). Outcome after pancreatectomy for neuroendocrine neoplasms. Clin Res Hepatol Gastroenterol.

[4] (2019). WHO Classification of Tumours Editorial Board.

[5] Acher AW, Hallet J (2024). Advances in Management of Nonfunctional Pancreas Neuroendocrine Tumors. Surg Clin North Am.

[6] Figueira ERR, Montagnini AL, Okubo J (2025). Non-functioning sporadic pancreatic neuroendocrine tumor is an independent risk factor for recurrence after surgical treatment. ABCD Arq Bras Cir Dig.

[7] Gavrilescu MM, Hutanu I, Scripcariu DV (2025). The Treatment of Pancreatic Neuroendocrine Tumors - A Retrospective Single-Centre Study. Chirurgia.

[8] Belotto M, Crouzillard B, Araujo KO (2023). Pancreatic neuroendocrine tumors: Surgical resection. ABCD Arq Bras Cir Dig.

[9] Regenet N, Carrere N, Boulanger G (2016). Is the 2cm size cutoff relevant for small nonfunctioning pancreatic neuroendocrine tumors: A French multicenter study. Surgery.

[10] Souche R, Hobeika C, Hain E, Gaujoux S (2020). Surgical Management of Neuroendocrine Tumours of the Pancreas. J Clin Med.

[11] Bader A, Landau S, Hwang J (2024). Recurrence and treatment trends of pancreatic neuroendocrine tumors. J Surg Oncol.

[12] Strosberg JR, Caplin ME, Kunz PL (2021). 177LuDotatate plus longacting octreotide versus highdose octreotide in patients with midgut neuroendocrine tumours (NETTER1): overall survival and longterm safety results from an openlabel, randomised, controlled, phase 3 trial. Lancet Oncol.

[13] KosKudła B, Castaño JP, Denecke T (2023). European Neuroendocrine Tumour Society. ENETS guidance paper for nonfunctioning pancreatic neuroendocrine tumours. J Neuroendocrinol.

[14] Vinik AI, Woltering EA, Warner RR (2010). North American Neuroendocrine Tumor Society. NANETS consensus guidelines for the diagnosis of neuroendocrine tumors. Pancreas.

